# E-cadherin to P-cadherin switching in lobular breast cancer with tubular elements

**DOI:** 10.1038/s41379-020-0591-3

**Published:** 2020-06-22

**Authors:** Matthias Christgen, Stephan Bartels, Jana L. van Luttikhuizen, Janin Bublitz, Luisa U. Rieger, Henriette Christgen, Helge Stark, Bjoern Sander, Ulrich Lehmann, Doris Steinemann, Patrick W. B. Derksen, Hans Kreipe

**Affiliations:** 1 Institute of Pathology, Hannover Medical School, Hannover, Germany; 2 Department of Human Genetics, Hannover Medical School, Hannover, Germany; 3 Department of Pathology, University Medical Center Utrecht, Utrecht, The Netherlands

**Keywords:** Breast cancer, Cell adhesion

## Abstract

Loss of E-cadherin expression due to mutation of the *CDH1* gene is a characteristic feature of invasive lobular breast cancer (ILBC). Beta-catenin, which binds to the cytoplasmic domain of E-cadherin, is simultaneously downregulated, reflecting disassembly of adherens junctions (AJs) and loss of cell adhesion. E-cadherin to P-cadherin expression switching can rescue AJs and cell adhesion. However, P-cadherin has not been implicated in ILBC, so far. We aimed to characterize 13 ILBCs with exceptional histomorphology, which we termed ILBCs with tubular elements. The *CDH1* mutational status was determined by next generation sequencing and whole-genome copy number (CN) profiling. Expression of cadherins was assessed by immunohistochemistry. ILBCs with tubular elements were ER-positive (13/13) and HER2-negative (13/13) and harbored deleterious *CDH1* mutations (11/13) accompanied by loss of heterozygosity due to deletion of chromosome 16q22.1 (9/11). E-cadherin expression was lost or reduced in noncohesive tumor cells and in admixed tubular elements (13/13). Beta-catenin expression was lost in noncohesive tumor cells, but was retained in tubular elements (11/13), indicating focal rescue of AJ formation. N-cadherin and R-cadherin were always negative (0/13). Strikingly, P-cadherin was commonly positive (12/13) and immunoreactivity was accentuated in tubular elements. Adjacent lobular carcinoma in situ (LCIS) was always P-cadherin-negative (0/7). In a reference cohort of LCIS specimens, P-cadherin was constantly not expressed (0/25). In a reference cohort of invasive mammary carcinomas, P-cadherin-positive cases (36/268, 13%) were associated with triple-negative nonlobular breast cancer (*P* < 0.001). Compared with ILBCs from the reference cohort, P-cadherin expression was more common in ILBCs with tubular elements (12/13 *versus* 7/84, *P* < 0.001). In summary, E-cadherin to P-cadherin switching occurs in a subset of ILBCs. P-cadherin is the molecular determinant of a mixed-appearing histomorphology in ILBCs with tubular elements.

## Introduction

Classical cadherins are transmembrane proteins that mediate homotypic adhesive cell–cell contacts by formation of adherens junctions (AJs) [1, 2]. They are implicated in a variety of cellular processes, such as cell polarization and differentiation [3]. Classical cadherins include epithelial (E-) and placental (P-) cadherin, which are encoded by the genes *CDH1* and *CDH3*, respectively [2]. Both map to chromosome 16q22.1 and share 66% homology [4, 5]. The luminal mammary epithelium is positive for E-cadherin, whereas myoepithelial mammary cells express P-cadherin [6, 7]. Classical cadherins play different roles in breast cancer (BC) [2]. E-cadherin functions as a tumor suppressor in invasive lobular breast cancer (ILBC) [8–10]. E-cadherin inactivation by somatic mutation of the *CDH1* gene drives development of lobular carcinoma in situ (LCIS) and ILBC [11, 12]. Beta-catenin, which binds to the cytoplasmic domain of E-cadherin, is simultaneously downregulated, reflecting disassembly of AJs and loss of cell adhesion in LCIS and ILBC [13, 14]. P120-catenin, another E-cadherin interaction partner, relocates from the cell membrane to the cytosol and nucleus [14]. In contrast to E-cadherin, P-cadherin expression is upregulated in triple-negative breast cancer (TNBC) of no special histological type (NST) and promotes tumor growth in this specific cellular context [7, 15]. Accordingly, P-cadherin is associated with poor prognosis in BC [7, 15].

Cadherin switching is a physiological process, in which cells shift to express different cadherins [1]. Cadherin switching regulates organ morphogenesis and tissue differentiation. For instance, E-cadherin to N-cadherin switching regulates primitive streak formation [1]. Aberrant cadherin switching has been observed in a variety of cancers and has profound effects on tumor phenotypes [1]. For instance, E-cadherin to N-cadherin switching is a feature of epithelial to mesenchymal transition [16]. N-cadherin to E-cadherin switching has been implicated in the progression of ovarian carcinomas [17]. E-cadherin to P-cadherin switching has been described for transitional cell carcinomas of the bladder [18]. The biological effects and regulation of cadherin switching are diverse and dependent on the specific cellular context [1].

Several experimental models indicate, that E-cadherin to P-cadherin switching can partially rescue AJ formation and cell–cell adhesion in the absence of E-cadherin [15, 19, 20]. Tinkle et al. targeted loss of E-cadherin in the mouse epidermis using conditional knockout (KO). E-cadherin KO induced only minor histological changes. Epidermal cells responded by upregulating P-cadherin, which partially rescued AJ formation and epithelial integrity [19]. However, terminal differentiation was impaired, resulting in hyperproliferative skin lesions [19]. Sarrio et al. and Ribeiro et al. determined that P-cadherin can function as a substitute for E-cadherin in TNBC. In their studies, ectopic expression or siRNA-mediated knockdown was used to obtain TNBC cell lines with E-cadherin negative but P-cadherin positive phenotypes [15, 20]. In the absence of E-cadherin, P-cadherin expression alone induced a relocation of beta-catenin to the cell membrane, rescued AJs formation, restored cell adhesion and reestablished a cohesive growth pattern in cell cultures [15, 20].

P-cadherin has not been implicated in the pathology or tumorbiology of ILBC [7, 15, 21]. In this study, we aimed to characterize a series of ILBCs with an exceptional, mixed-appearing (cohesive/noncohesive) histomorphology, which we termed “ILBCs with tubular elements.” Expression of alternate cadherins was determined by immunohistochemistry. Our findings reveal that E-cadherin to P-cadherin switching is the molecular determinant of these ILBCs with tubular elements.

## Materials and methods

### Tumor specimens

Tumor tissues included formalin-fixed paraffin-embedded (FFPE) specimens of *n* = 13 patients diagnosed with ILBC at the Institute of Pathology at the Hannover Medical School (MHH) (Table 1). Cases were collected over a period of 2 years and were selected from *n* = 958 consecutive BC cases encountered in routine clinical diagnostics during that time. Inclusion criteria were as follows: (1) consensus on BC subtype classification as ILBC (with noncohesive tumor cells arranged in conventional ILBC growth pattern, such as classical, dispersed, trabecular, and solid growth pattern) among ≥3 pathologists specialized in breast pathology (based on assessment of hematoxylin/eosin-stained sections), (2) consensus on the presence of focal tubular elements showing similar cytological and nuclear features, (3) negative or aberrant E-cadherin immunoreactivity, and (4) consensus that tubular elements showed negative or aberrant E-cadherin immunoreactivity too. All cases were discussed in detail on a multi-headed microscope before inclusion in this study. Cases that did not meet consensus criteria were not included. Further FFPE specimens included *n* = 25 LCIS lesions from *n* = 19 patients and tissue microarrays (TMAs, 1.5 mm core diameter) of *n* = 268 invasive BCs, which served as reference cohorts [22]. FFPE tissue specimens were retrieved from the archives of the Institute of Pathology at the MHH in accordance with the guidelines of the local ethics committee (MHH, Hannover, Germany). All specimens were made anonymous for scientific purposes.

**Table 1:** Clinicopathological characteristics.

Case	Age	Tissue	Histology, grade	Growth pattern	LCIS	pTNM	ER	PR	HER2	HER2 FISH	Ki67 (%)	E-cad.	beta- ctn.	CDH1 status	LOH 16q22.1
1	69	RS	ILBC-TE, G3	sol, trab, te (60%)	LCIS	pT2m, pN0	pos	neg	2+	neg	20	neg	fpos	p.H97Ifs*20	LOH
2	79	RS	ILBC-TE, G2	sol, clas, te (20%)	LCIS	pT1c, pNx	pos	pos	1+	na	20	neg	fpos	p.F86Sfs*12	LOH
3	64	RS	ILBC-TE, G2	sol, clas, te (60%)		rpM1 (ovary)	pos	pos	1+	na	20	neg	fpos	p.S9*	cnLOH
4	50	NB	ILBC-TE, G2	trab, disp, te (20%)	LCIS	pTxmBi, pNx	pos	pos	2+	neg	15	faint	fpos	p.E841*, p.Y523*	na
5	61	RS	ILBC-TE, G2	sol, trab, te (15%)	LCIS	pT1cm, pN0	pos	pos	1+	na	15	neg	fpos	p.Q23*	cnLOH
6	56	NB	ILBC-TE, G2	clas, te (20%)		pT1b, pN0	pos	neg	1+	na	20	neg	neg	wt	LOH
7	58	NB	ILBC-TE, G2	clas, disp, te (15%)	LCIS	pT2, pN0	pos	pos	1+	na	20	neg	fpos	p.Q177*	LOH
8	50	NB	ILBC-TE, G2	trab, te (5%)		pT2, pN1mi	pos	pos	1+	na	20	neg	neg	p.E445*	LOH
9	42	RS	ILBC-TE, G2	clas, te (60%)	LCIS	pT2m, pN1	pos	pos	1+	na	15	neg	fpos	Splicing	LOH
10	60	RS	ILBC-TE, G2	clas, te (40%)		pT2, pN0	pos	pos	2+	neg	20	neg	fpos	p.T295I	na
11	59	RS	ILBC-TE, G2	clas, te (20%)		pT1c, pN1mi	pos	pos	1+	na	15	neg	fpos	p.P537Rfs*20	LOH
12	75	NB	ILBC-TE, G2	clas, te (25%)		pT3, pN1	pos	pos	1+	na	20	neg	fpos	p.Q23*	LOH
13	48	RS	ILBC-TE, G3	clas, trab, te (15%)	LCIS	pT1c, pN1	pos	pos	1+	na	25	neg	fpos	wt	LOH

*Beta-ctn* beta-catenin, *Bi* bilateral, *clas* classical growth pattern, *cn* copy number-neutral, *E-cad.* E-cadherin, *fpos* focally positive, *ILBC-TE* invasive lobular breast cancer with tubular elements, *LCIS* lobular carcinoma in situ, *LOH* loss of heterozygosity, *NB* needle biopsy, *neg* negative, *pos* positive, *RS* resection specimen, *sol* solid growth pattern, *trab* trabecular growth pattern, *te* (%) tubular elements (% tumor area), *wt* wild-type.

### Immunohistochemistry

For immunohistochemistry, 1 µm thick sections of FFPE tissue blocks or TMAs were mounted on superfrost slides (Thermo Fisher Scientific, Rockford, USA). Next, slides were deparaffinized and rehydrated conventionally and were subjected to immunohistochemical staining using a Benchmark Ultra (Ventana, Tucson, USA) automated stainer. The CC1 mild program was used for antigen retrieval and the ultraView DAB kit (Ventana) for signal detection. Antibodies used for immunohistochemistry included the monoclonal anti-E-cadherin antibody ECH-6 (1:100, Zytomed, Berlin, Germany), the monoclonal anti-beta-catenin antibody clone 14 (1:75, BD Transduction Laboratories, Franklin Lakes, USA) the monoclonal anti-P-cadherin antibody clone 56 (1:100, BD Transduction Laboratories), the monoclonal anti-N-cadherin antibody 3B9 (1:100, Thermo Fisher Scientific, Rockford, USA) and the monoclonal anti-R-cadherin antibody D-9 (1:50, Santa Cruz, Santa Cruz, USA) Further antibodies are described in Supplementary data Table 1 (Supplementary data Table 1). Membranous P-cadherin immunoreactivity was scored using an immunoreactivity score (IRS) as described by Remmele and Stegner [22, 23]. Immunohistochemistry scoring was discussed on a multi-headed microscope until consensus was achieved among ≥3 pathologists.

### Automated Ki67 quantification

Ki67-stained sections of ILBCs were scanned with an Aperio CS2 histological slide scanner at ×200 magnification (Leica Biosystems, Wetzlar, Germany). Percentages of Ki67-stained tumor cells per field of view (FOV, 0.36 mm^2^ size) were quantified with CognitionMaster Professional Suite (Vmscope, Berlin, Germany) [24]. For each case, multiple FOVs with either conventional growth pattern or tubular elements were analyzed. For conventional growth pattern, the median number of FOVs per case was 12 (range 6–28). The median tumor cell count per case was 7603 (range 2820–24,454). The total number of FOVs in all specimens was 145 and the total tumor cell count was 110,123. For tubular elements, the median number of FOVs per case was 5,5 (range 4–26). The median tumor cell count per case was 1082 (range 250–7065). The total number of FOVs in all specimens was 85 and the total tumor cell count was 21,481.

### DNA extraction

Genomic DNA was extracted as described previously [25]. In brief, tumor tissue was marked on HE-stained sections of FFPE tissue blocks. Corresponding tissue areas were microdissected with a surgical blade on unstained sections (*n* = 10, 8 µm each) from this FFPE tissue block. Another HE stain prepared after cutting of the unstained sections confirmed unaltered tumor representation on deeper sections of the tissue blocks. For large tumor specimens, DNA was extracted from bulk tissue. For needle biopsies, microdissection of tumor tissue was omitted as well. Next, genomic DNA was extracted with the Maxwell RSC DNA FFPE kit (Promega, Madison, USA) on a Maxwell RSC instrument (Promega) according to the manufacturer’s recommendations. DNA was quantified using a Qubit 2.0 fluorometer (Invitrogen) and the Qubit dsDNA HS assay kit (Life Technologies, Carlsbad, USA).

### Mutational analysis

Mutational analysis of the *CDH1* gene was carried out by next generation sequencing (NGS) as described previously [25, 26]. NGS was performed with genomic DNA on an Ion S5 system (Life Technologies, Carlsbad, USA) using a customized *CDH1* NGS panel designed with Ion AmpliSeq^TM^ designer software (version 5.6). This panel covered the complete protein-coding sequence of the *CDH1* gene (16 exons, 882 codons), the 5′-UTR sequence of exon 1 and the 3′-UTR sequence of exon 16 with 26 amplicons [25]. Mean (*n* = 21) mapped reads per sample was 517,676 (range 35,952 to 1,891,618), mean depths per base was 15,895 (range 857 to 57,331) (Supplementary data Table 2). Amplicons passed QC parameters when the amplicon coverage was at least 500, with a minimum of 100 reads per sequencing direction. Variants were required to have an allelic frequency of at least 3% to be considered as true positives. Variant annotation was performed with the ANNOVAR analysis software and database tools (http://www.openbioinformatics.org/annovar) [27]. BAM files were preanalyzed with the Ion Reporter software (version 5.10.1.0, Thermo Fisher Scientific). One case (case 3, ILBC metastatic to the ovary, two regions) was also analyzed with the Oncomine comprehensive assay (Thermo Fisher Scientific), covering variants across 161 genes, CNVs and gene fusions. Mapped reads were 9,907,247 and 5,562,786 (mean coverage 2856 and 1587) per sample. Variant annotation was performed as described above.

### DNA copy number profiling

Whole-genome DNA copy number (CN) profiling was performed using molecular inversion probe (MIP) arrays (OncoScan^TM^, Affymetrix, Santa Clara, CA, USA) and 80 ng total DNA, as described previously [25]. OSCHP files were produced from the CEL files by Chromosome Analyses Suite (ChAS) software (version 4.0.0.385). If necessary, sample data were recentered manually, based on allele differences, B-allele frequencies (BAFs) and weighted log_2_-ratios. The MIP array data series is deposited in the Gene Expression Omnibus database (GSE134844 submitted 25.07.2019). Weighted log_2_-ratios and BAFs of all samples were extracted from the ChAS software and were subsequently analyzed with the R packages “copynumber” (version 1.18.0) [28] and “Clonality” (version 1.26.0) [25, 29, 30]. For allele-specific CN segmentation the “copynumber::aspcf” method was used, with default settings. The thresholds used to call losses and gains were −0.1 and 0.1, respectively. Clonal relatedness of CN profiles was determined using the likelihood ratio (LR) method “Clonality::clonality.analysis” (parameter “nmad” = 1.0) after averaging of adjacent probes (parameter “K” = 2). The LR quantifies the odds that two given tumors are clonal and is benchmarked against the distribution of LRs in pairs of independent tumors from independent patients in a reference cohort [29].

### Quantitative real-time RT-PCR

For assessment of gene expression, RNA was extracted from microdissected tumor tissue using the Maxwell RSC RNA FFPE kit (Promega) on a Maxwell RSC instrument (Promega) according to the manufacturer’s recommendations. RNA concentrations were quantified using a Qubit 2.0 fluorometer (Invitrogen, Darmstadt, Germany) and the Qubit RNA high sensitivity assay kit (Life Technologies, Carlsbad, CA, USA). Five hundred nanograms RNA input was used for cDNA synthesis with the SuperScript^®^ VILO cDNA synthesis kit (Invitrogen). For single qPCR reactions, 25 ng cDNA was used and all measurements were performed in technical triplicates per target. Quantitative real-time PCR for *CDH1*, *CDH3*, and the two housekeeping genes *TBP* and *GUSB* was carried out with specific PCR primers on an ABI 7500 real-time PCR instrument (Applied Biosystems, ThermoFisher Scientific, Waltham, MA, USA). Exon junction spanning TaqMan^®^ probes (ThermoFisher Scientific, Waltham, MA, USA) for *CDH1* (Hs01013958_m1), *CDH3* (Hs00999915_m1), *TBP* (Hs00427620_m1), and *GUSB* (Hs00939627_m1) were used for detection of PCR products. Gene expression was normalized to two housekeeping genes (*GUSB* and *TBP*). Statistical significance of normalized gene expression level differences was assessed with the unpaired *t*-test and GraphPad Prism software (version 5.00, La Jolla, CA, USA).

### TCGA RNAseq expression data

The cancer genome atlas (TCGA) data set “Breast Invasive Carcinoma” (Ciriello et al.) was extracted from cBioPortal (http://download.cbioportal.org/brca_tcga_pub2015.tar.gz) [31]. This data set provides *CDH1* (E-cadherin) and *CDH3* (P-cadherin) expression data from RNA sequencing (RNAseq) [31]. Normalized and log2-transformed RNAseq by expectation maximation data, reflecting relative mRNA expression, were retrieved from the “data_RNA_Seq_v2_expression_median.txt” file. Based on TCGA sample annotations, a refined re-analysis was performed for four BC subsets including: (1) TNBC of NST, (2) hormone receptor (HR)-positive BC of NST, and (3) ILBC (*CDH1* mutation positive).

### Statistics

Statistical significance of different proportions of P-cadherin-positive and P-cadherin-negative cases in ILBCs from the reference cohort and ILBCs with tubular elements was assessed with Fisher’s exact test using GraphPad Prism software (version 5.0, La Jolla, USA). Significance of different Ki67 indices per FOV in ILBC cells arranged in conventional growth pattern versus tubular elements was determined with the Mann–Whitney test and GraphPad Prism software.

## Results

### Clinicopathological characteristics

**Fig. 1:**
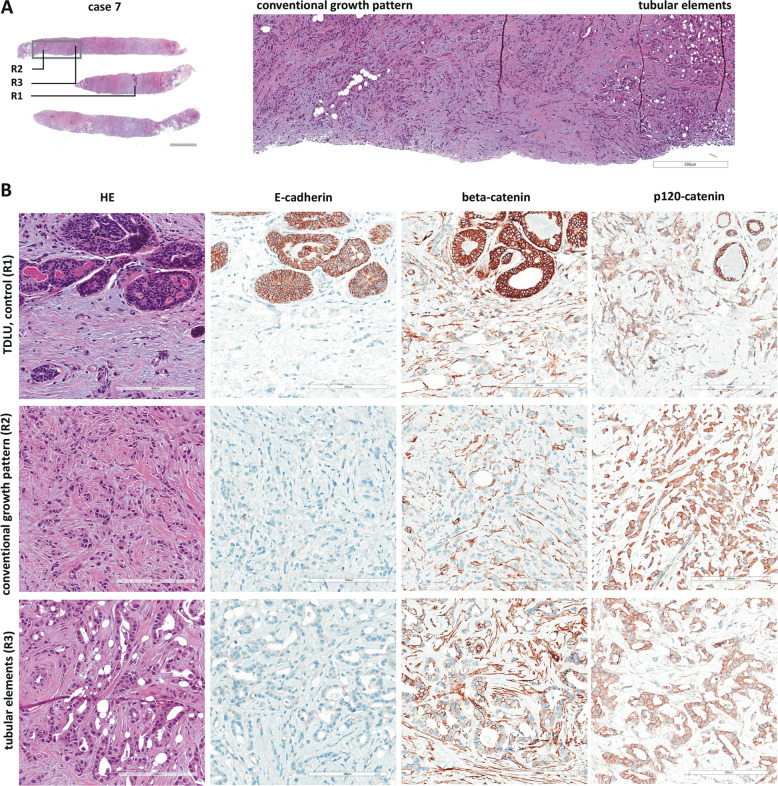
Histology of ILBC with tubular elements. Shown is a representative specimen (case 7). **a** HE-stained biopsy (left, scale bar corresponds to 2 mm) and submacroscopic view (right, ×50 magnification, scale bar corresponds to 500 µm). The gray rectangle indicates the area shown in the submacroscopic view. Labels R1-3 indicate regions shown in detail. **b** Details from regions R1 (TDLU terminal ductolobular unit, internal control), region R2 (conventional ILBC growth pattern), and R3 (tubular elements). Photomicrographs of immunohistochemical stainings for E-cadherin, beta-catenin and p120-catenin from consecutive serial sections are also provided (×200 magnification, scale bar corresponds to 200 µm).

In this study, we aimed to characterize a series of 13 ILBCs with unusual histomorphology (Table 1). Cases selected for this study were defined by noncohesive tumor cells arranged in conventional ILBC growth pattern (such as classical, dispersed, trabecular, and solid growth pattern, which are reviewed elsewhere [10]) admixed with well-defined cancerous tubular elements (Fig. 1). In admixed tubular elements, tumor cells appeared to gain cell adhesion. Tubular elements displayed variable size and shape (Supplementary data Fig. 1). Four cases showed longitudinal or tear drop-shaped tubular elements, partly reminiscent of G1-differentiated tubular carcinomas (cases 4, 11, 12, 13). Another four cases showed small round tubules with narrow lumina (cases 5, 7, 8, 10). Tubular elements occupied ~20% of tumor areas (range 5–60%) (Table 1). Nuclear atypia was commonly nuclear grade 1/2. Cytological and nuclear features were similar in noncohesive tumor cells and admixed tubular elements. We termed these exceptional cases “ILBC with tubular elements.” Median patient age was 59 years (range 42–79 years). Synchronous LCIS was associated with 7/13 (54%) cases. Bifocal or multifocal BC was evident in 4/13 (31%) cases. One case (case 4) was a bilateral BC with two tumors in the left breast and one tumor in the right breast. In bifocal, multifocal and bilateral BCs, all additional tumors were conventional ILBCs without tubular elements. One case (case 3) was an ovarian metastasis in a female patient with a history of ILBC (Table 1).

### Loss of E-cadherin but not beta-catenin

**Fig. 2:**
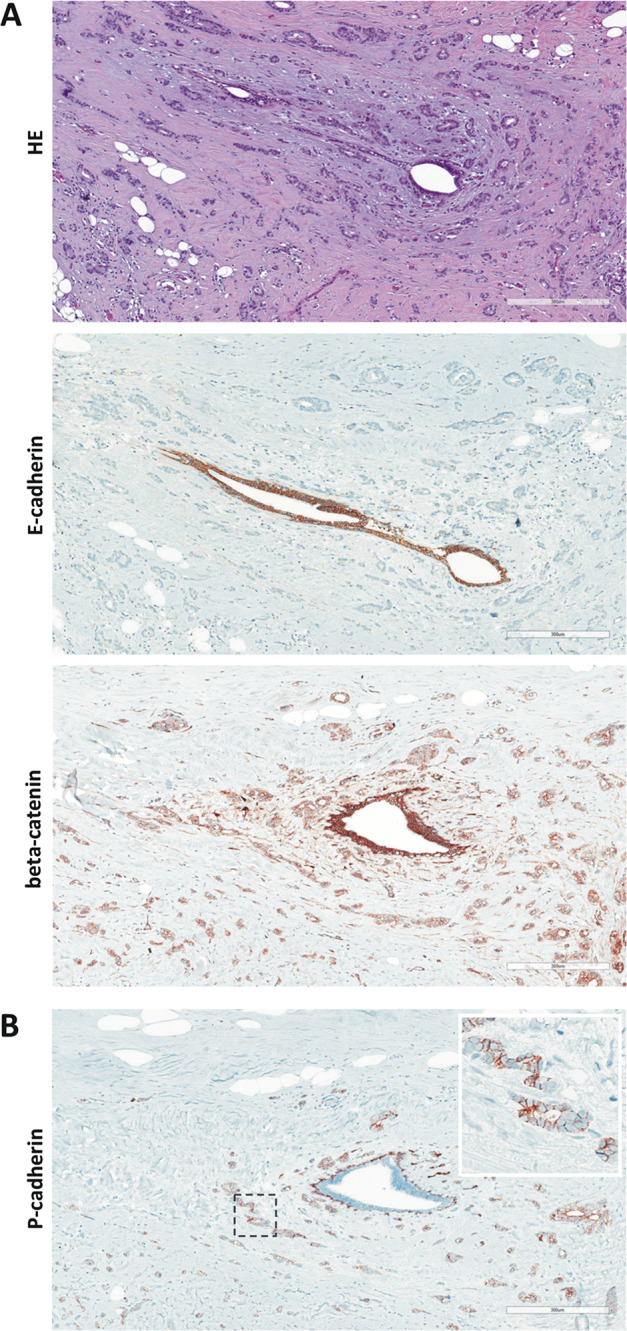
Histology of ILBC with tubular elements. Shown is a representative specimen (case 11). E-cadherin-negative tubular elements spread around a mammary duct. **a** The upper panel shows details from the HE-stained section (×100 magnification, scale bar corresponds to 300 µm). Photomicrographs of immunohistochemical stainings for E-cadherin and beta-catenin from consecutive serial sections are also provided (×100 magnification, lower panels). Please note that cancerous tubular elements lack E-cadherin immunoreactivity, while the normal mammary duct in the center stains positive for E-cadherin. Note that beta-catenin immunoreactivity in cancerous tubular elements was weaker than in the normal mammary duct. **b** Immunohistochemical staining for P-cadherin from a consecutive serial section. Please note the same normal mammary duct in the center. The normal mammary duct shows regular immunoreactivity for P-cadherin confined to the myoepithelial cell layer. Cancerous tubular elements spread around this duct are positive for P-cadherin (inset).

All cases were subjected to immunohistochemical characterization (Table 1). In total, 13/13 (100%) cases were estrogen receptor (ER)-positive and HER2-negative. Complete loss of E-cadherin immunoreactivity was observed in 12/13 (92%) cases. Tubular elements were E-cadherin-negative too (Fig. 1). One case (case 4) showed strongly reduced E-cadherin immunoreactivity, also in tubular elements. Adjacent normal mammary gland ducts showed regular E-cadherin immunoreactivity, which verified appropriate immunohistochemical staining (Fig. 2a). Loss of E-cadherin is typically accompanied by loss of beta-catenin expression and cytoplasmic mislocalization of p120-catenin in ILBC [13, 14]. Beta-catenin binds to the cytoplasmic region of E-cadherin and links AJs to the actin cytoskeleton. Downregulation of beta-catenin protein reflects the disassembly of AJs in the absence of E-cadherin [13, 14]. Remarkably, 11/13 (85%) cases lacked E-cadherin but retained focal membranous beta-catenin expression (Table 1). In particular, tubular elements were E-cadherin-negative and beta-catenin positive (Fig. 1). Due to limited tissue, p120-catenin was not studied in all cases. However, membrane-localized p120-catenin expression was noted in tubular elements in some cases (cases 3 and 7), but was not unambiguously seen in other cases (cases 9 and 11) (Fig. 1). Compared with adjacent normal ducts, beta-catenin staining intensity was slightly weaker in cancerous tubular elements (Fig. 2). Hence, ILBCs with tubular elements showed complete loss of E-cadherin but retained membrane-localized beta-catenin expression, indicating focal rescue of AJ formation.

### Common *CDH1*/E-cadherin mutations

**Fig. 3:**
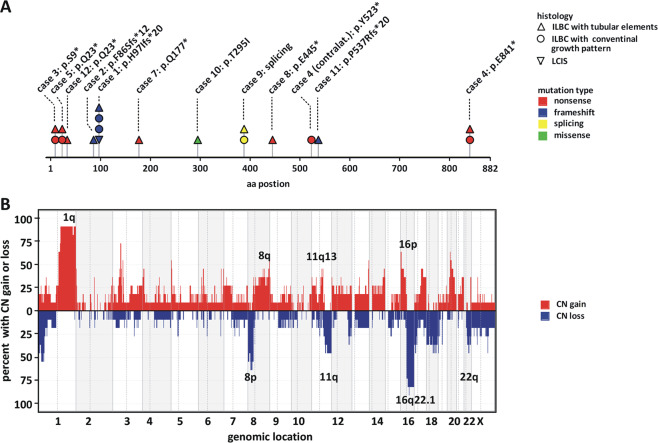
*CDH1* mutation and loss of chromosome 16q. **a** Shown is the protein-coding sequence of the *CDH1*/E-cadherin gene. Lesion histology is coded by the symbol shape, as indicated in the legend. Mutation type is coded by the symbol color, as indicated in the legend. For details see Supplementary data Table 2. **b** Genome-wide view of CNAs detected in ILBCs with tubular elements (*n* = 11) based on whole-genome DNA CN profiles. Chromosomal localization is plotted on the *x* axis. The overall frequency of CN gains (red) and CN loss (blue) is plotted on the *y* axis.

To substantiate the loss of functional E-cadherin, all cases were subjected to mutational analysis of *CDH1* (Fig. 3a). *CDH1* mutations were detected in 11/13 (85%) cases. Six cases (cases 3, 4, 5, 7, 8, 12) harbored nonsense mutations introducing premature termination codons. An additional three cases (cases 1, 2, 11) harbored frameshift mutations introducing premature termination codons. One case (case 9) showed a splice site mutation. In addition, one case (case 10) harbored a missense mutation. Two cases (cases 6 and 13) harbored no detectable *CDH1* mutation. Interestingly, case 4 harbored a nonsense mutation (p.E841*) in the C-terminal cytoplasmic region. This mutation site is located downstream of the nonsense-mediated decay boundary, which is relevant for RNA surveillance mechanisms that suppress translation of aberrant mRNAs [32]. This correlated well with the strongly reduced E-cadherin immunoreactivity observed in this particular tumor (Table 1).

Four cases (case 1, 4, 5, 9) were bifocal, multifocal, or bilateral BCs. Additional tumors from these patients were conventional ILBCs without tubular elements. For completeness, we also analyzed these additional lesions for *CDH1* mutations. *CDH1* mutations were always identical in conventional ILBCs, LCIS, and ipsilateral ILBCs with tubular elements, indicating clonal relatedness (Fig. 3a). For instance, case 1 featured three invasive BCs (two ILBCs with conventional growth pattern, one ILBC with tubular elements) and an adjacent LCIS. All four lesions, including the LCIS, shared the same *CDH1* mutation (p.H97Ifs*20) (Fig. 3a). Case 4 featured two BCs in the left breast (one ILBC with conventional growth pattern, one ILBC with tubular elements) and one ILBC in the right breast (ILBC with conventional growth pattern). The two ILBCs in the left breast shared the same *CDH1* mutation (p.E841*), indicating clonal relatedness. The ILBC in the right breast harbored a different *CDH1* mutation (p.Y523*), indicating clonal independence. Moreover, case 3 (ILBC metastatic to the ovary) featured regions with pure conventional ILBC growth pattern and other regions with almost pure tubular elements. Tumor tissue from these regions was purified by microdissection and was subjected to *CDH1* mutational analysis. As expected, tumor tissue from both growth patterns harbored the same *CDH1* mutation (p.S9*) and showed similar allelic burden (0.75 and 0.70, see below) (Supplementary data Table 2). The overall *CDH1* mutation spectrum was consistent with previous studies [9]. Four *CDH1* mutations described here (p.S9*, p.Q23*, p.Y523*, and p.E841*) have been reported for ILBC earlier [9]. Hence, deleterious *CDH1* mutations were common in ILBCs with tubular elements.

### Common loss of chromosome 16q22.1

Inactivation of *CDH1* is typically caused by heterozygous mutation and subsequent loss of heterozygosity (LOH) [9]. Whole-genome DNA CN profiles were obtained from 11/13 ILBCs with tubular elements. In two cases (case 4 and 10) CN profiling was not possible due to limited DNA amount. Recurrent CN alterations (CNAs) included gains on chromosome 1q, 8q, 11q13, and 16p and losses on chromosome 8p, 11q, 16q, and 22q (Fig. 3b). This is consistent with previously published ILBC series [9, 33–35]. CN losses and LOH of chromosome 16q, including 16q22.1 (*CDH1* gene locus), were detected in 9/11 (82%) cases. The remaining cases (cases 3 and 5) harbored CN-neutral LOH (cnLOH) of chromosome 16q22.1, as determined by B-allele frequency analysis (Supplementary data Fig. 2A). Two ILBCs without a detectable *CDH1* mutation (cases 6 and 13) displayed CN losses and LOH of chromosome 16q22.1 (Supplementary data Fig. 2B). However, a CN < 1.0 of chromosome 16q22.1, indicative of a homozygous deletion of the *CDH1* gene locus, was not detected. Taken together, 11/11 (100%) cases harbored either a CN loss and LOH or a cnLOH of the *CDH1* gene locus (Table 1).

### Clonal relatedness to adjacent conventional ILBC

Four additional tumors from cases 1, 5, and 9, corresponding to adjacent conventional ILBCs or LCIS, were also subjected to CN profiling. Moreover, microdissected tumor tissue with only conventional ILBC growth pattern from case 3 was subjected to CN profiling. CN profiles of these additional lesions matched with CN profiles of adjacent ILBCs with tubular elements indicating clonal relatedness (Supplementary data Fig. 3). To analyze clonal relatedness more comprehensively, we employed the statistical LR method [29, 30]. The LR method determines clonality or independence of two tumors based on the overall pattern of CNAs [29, 30]. The complete series of ILBCs with tubular elements and adjacent lesions (*n* = 16 samples) served as a reference cohort, providing *n* = 114 nonclonal tumor pairs from independent patients for this statistical analysis. LR values, reflecting the odds that two tumors are clonal, ranged from 3 × 10^−8^ to 2 × 10^8^ (median 2 × 10^−4^) in tumors from independent patients (reference) (Supplementary data Fig. 3). LRs for ILBCs with tubular elements and adjacent conventional ILBCs or LCIS ranged from 3.3 × 10^5^ to 6.1 × 10^37^, which formally proved clonal relatedness of adjacent lesions in individual patients (1 × *P* = 0.009, 5 × *P* < 0.001) (Supplementary data Fig. 3). Hence, ILBCs with tubular elements and adjacent ILBCs with conventional growth pattern were essentially different morphological presentations of tumor cells sharing the same clonal ancestry.

### Common expression of P-cadherin

**Fig. 4:**
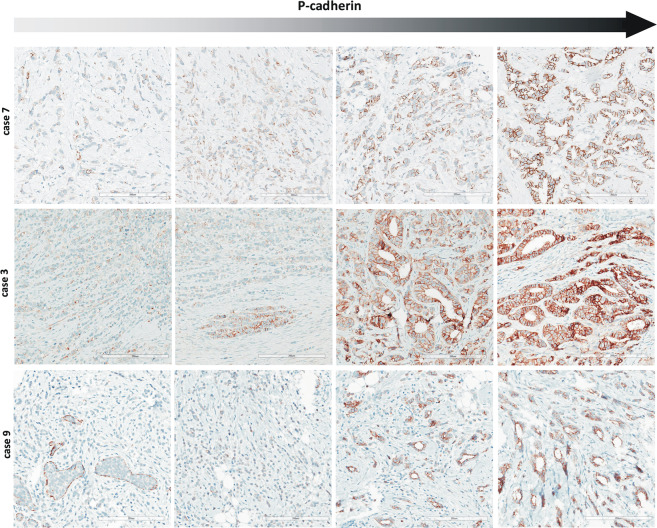
P-cadherin expression in ILBC with tubular elements. Shown are representative photomicrographs of P-cadherin immunohistochemical stainings from four different regions of case 7 (upper panels), case 3 (middle panel), and case 9 (lower panel) (×200 magnification, scale bars correspond to 200 µm). Please note that membranous immunoreactivity for P-cadherin is the strongest in tubular elements (right panels). Noncohesive tumor cells forming conventional ILBC growth pattern are mostly, but not exclusively P-cadherin-negative (left panels). Adjacent LCIS is P-cadherin-negative (lower left panel).

ILBCs with tubular elements showed complete loss of E-cadherin but retained membrane-localized beta-catenin expression, indicating focal rescue of AJs. We hypothesized, that this was due to activation of alternate cadherins [1, 19]. Accordingly, P-, N-, and R-cadherin expression was determined by immunohistochemistry (Table 2). Myoepithelial cells of normal mammary ducts served as positive control for appropriate P-cadherin staining (Supplementary data Fig. 4). Intercalating disks of myocard served as positive control for appropriate N- and R-cadherin staining (Supplementary data Fig. 4). None of the cases showed any specific staining for N- or R-cadherin (0/13, 0%). Strikingly however, P-cadherin was positive in 12/13 (92%) cases (Table 2). P-cadherin immunoreactivity was strongest in tubular elements (Fig. 4). Noncohesive tumor cells forming the conventional ILBC growth pattern showed weaker or no immunoreactivity. Adjacent normal mammary gland ducts showed regular P-cadherin immunoreactivity confined to the myoepithelial cell layer, which verified appropriate immunohistochemical staining (Fig. 2b). Thus, ILBCs with tubular elements displayed a distinctive heterogeneity or spatial gradient of P-cadherin expression (Fig. 4). In one specimen (case 10) P-cadherin-negative, noncohesive tumor cells appeared to bud from or associated with aggregates of P-cadherin-positive, cohesive tumor cells (Supplementary data Fig. 5). Depending on the number of tubular elements, overall P-cadherin IRS ranged between IRS 3 and IRS 9. Hence, ILBCs with tubular elements are commonly positive for P-cadherin.

**Table 2:** Classical (type-I) cadherin expression.

Case	P-cad.	N-cad.	R-cad.
1	fpos	neg	neg
2	fpos	neg	neg
3	fpos	neg	neg
4	fpos	neg	neg
5	fpos	neg	neg
6	fpos	neg	neg
7	fpos	neg	neg
8	neg	neg	neg
9	fpos	neg	neg
10	fpos	neg	neg
11	fpos	neg	neg
12	fpos	neg	neg
13	fpos	neg	neg

*Fpos* focally positive, *neg* negative, *pos* positive, *N-cad*. N-cadherin, *P-cad*. P-cadherin, *R-cad.* R-cadherin.

### *CDH3*/P-cadherin mRNA expression

**Fig. 5:**
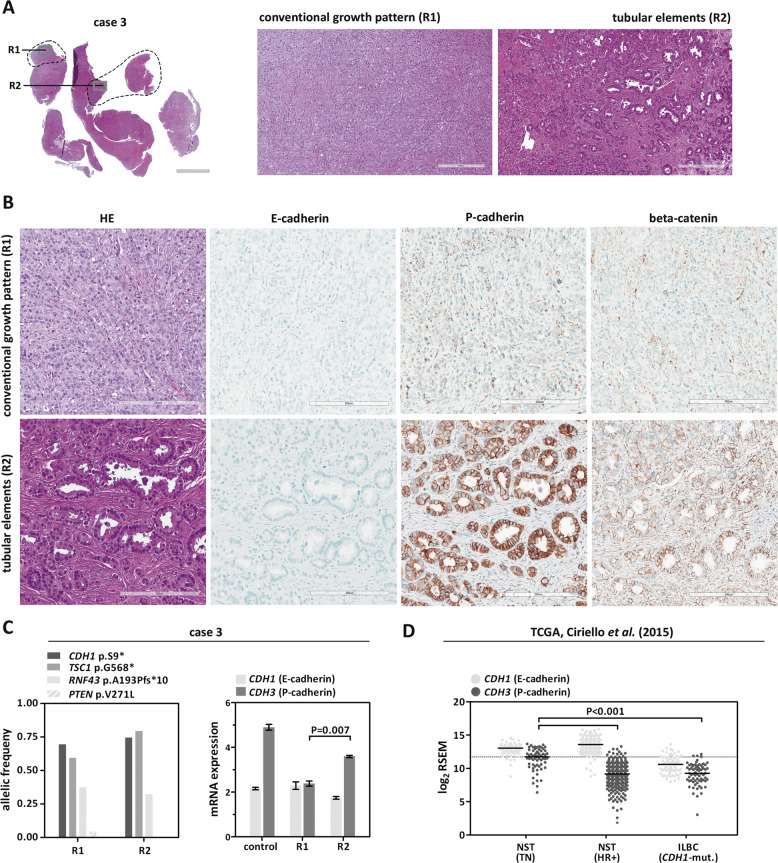
Histology of ILBC with tubular elements. Shown is representative specimen (case 3). **a** HE-stained section of an ILBC metastasis to the ovary, cut in slices (left, scale bar corresponds to 3 mm) and submacroscopic view (right, ×100 magnification, scale bar corresponds to 300 µm). The gray rectangle indicates areas shown in the submacroscopic view. Labels R1 and R2 indicate regions shown in detail. The dashed line indicates tumor areas subjected to microdissection for extraction of DNA and RNA. **b** Details from regions R1 (conventional growth pattern) and R2 (tubular elements). Photomicrographs of repeated immunohistochemical stainings for E-cadherin, P-cadherin, and beta-catenin from a second set of new consecutive serial sections (×200 magnification, scale bar corresponds to 200 µm). **c** Mutational analysis (left) and quantitative real-time RT-PCR (right). Expression of *CDH1* (E-cadherin) and *CDH3* (P-cadherin) was assessed in microdissected tumor tissue from regions R1 and R3. Shown is relative mRNA expression normalized to two housekeeping genes (*GUSB* and *TBP*). Error bars indicate SEM. Normal mammary tissue served as a control (independent patient). Statistical significance was determined with the unpaired *t*-test. **d** Re-analysis of TCGA gene expression data (Ciriello et al. [31]). Shown are normalized and log_2_-transformed RNAseq by expectation maximation (RSEM) data, reflecting relative mRNA expression. This re-analysis included *n* = 64 triple-negative BC of no special type (NST, TN), *n* = 327 hormone receptor-positive BC of no special type (NST, HR+), and *n* = 70 invasive lobular breast cancer with deleterious *CDH1* mutation (ILBC, *CDH1*-mut.). Each dot corresponds to an individual BC. Horizontal lines; median expression levels. Dashed line; median expression level in triple-negative BC/NST. Statistical significance was assessed with the Mann–Whitney test.

ILBCs with tubular elements were commonly P-cadherin positive. Immunohistochemical stainings for E-cadherin and P-cadherin were repeated using new consecutive serial sections from case 3 (ILBC metastatic to the ovary). This confirmed that tubular elements were E-cadherin negative, P-cadherin positive, and beta-catenin positive (Fig. 5a, b). Repeated immunohistochemical staining of cases 11 and 13 showed the same result (Supplementary data Figs. 6 and 7). Next, we assessed *CDH3* (P-cadherin) mRNA expression. Case 3 was chosen for this analysis for three reasons: first, case 3 lacked normal mammary tissue or other epithelia in the microenvironment. Second, this specimen showed a high tumor cellularity. Third, case 3 featured regions with pure conventional growth pattern (region R1) and regions with almost pure tubular elements (region R2). Tumor tissue from these regions was purified by microdissection for extraction of DNA and RNA. Then, DNA was subjected to mutational analysis by NGS (customized *CDH1* panel and Oncomine comprehensive assay), while RNA was subjected to quantitative real-time RT-PCR. Both regions harbored the same *CDH1/*E-cadherin mutation (p.S9*), the same *TSC1* mutation (p.G568*) and the same *RNF43* mutation (p.A193Pfs*10) with equally high allelic burden (Fig. 5c, left). This confirmed that microdissected regions harbored tumor cells sharing the same clonal ancestry. *CDH1* (E-cadherin) and *CDH3* (P-cadherin) mRNA were expressed in both regions, as determined by quantitative real-time RT-PCR. Compared to normal mammary tissue (control), *CDH3* (P-cadherin) mRNA was expressed at lower levels in the metastatic ILBC. Compared to region R1 (conventional growth pattern), region R2 (tubular elements) showed increased *CDH3* (P-cadherin) mRNA expression (*P* = 0.007) (Fig. 5c, right). Next, publicly available TCGA RNAseq data reported by Ciriello et al. were re-analyzed to validate *CDH3* (P-cadherin) expression in ILBCs from an independent tumor cohort [31]. As expected, *CDH3* mRNA expression levels were higher in TNBC of NST compared to HR-positive BCs of NST or ILBC (*P* < 0.001) (Fig. 5d). This is consistent with the known upregulation of P-cadherin in TNBC [7, 15]. Interestingly, 4/70 (6%) ILBCs included in this re-analysis showed *CDH3* mRNA expression levels above the median *CDH3* mRNA expression level in TNBC (Fig. 5d).

### Adjacent LCIS is P-cadherin-negative

**Fig. 6:**
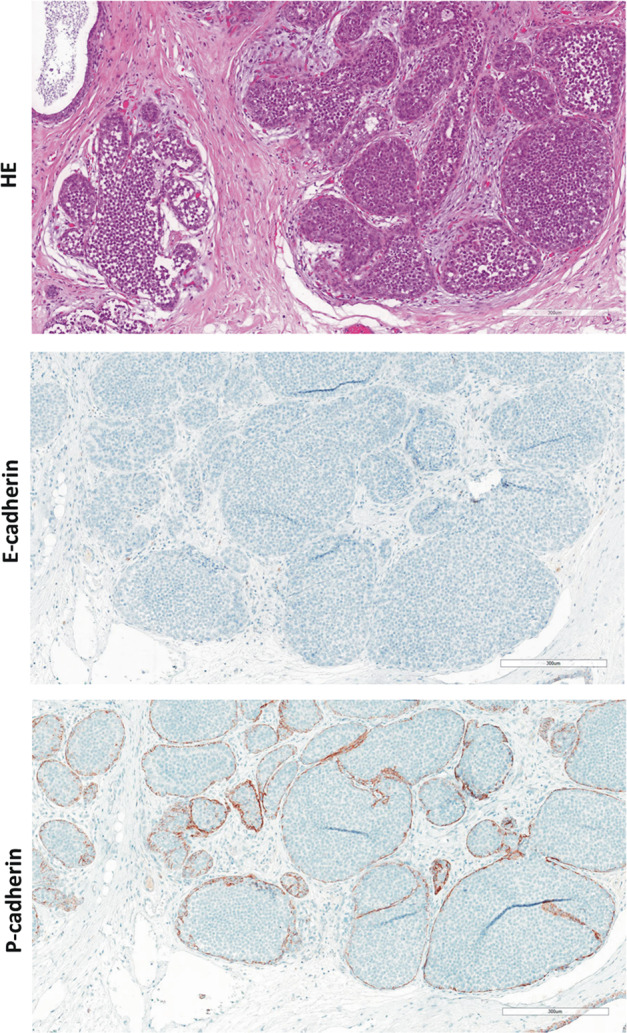
Lack of P-cadherin expression in LCIS. Shown is a representative photomicrograph of an LCIS specimen from the reference cohort. The upper panel shows details from the HE-stained section at ×100 magnification. Scale bar corresponds to 300 µm. Photomicrographs of immunohistochemical stainings for E-cadherin and beta-catenin are also provided (×100 magnification, lower panels).

LCIS is a nonobligate precursor of ILBC [11]. The series of ILBCs with tubular elements included *n* = 7 cases with synchronous LCIS. P-cadherin immunoreactivity of adjacent LCIS was observed in 0/7 cases (Fig. 4, lower left). To substantiate this, an independent reference cohort *n* = 25 LCIS specimens from *n* = 19 patients was subjected to P-cadherin immunohistochemistry. P-cadherin immunoreactivity was observed in 0/25 (0%) LCIS specimens (Fig. 6). Hence, LCIS is typically P-cadherin-negative, even if associated with a P-cadherin-positive ILBC with tubular elements.

### P-cadherin is associated with ILBCs with tubular elements

**Fig. 7:**
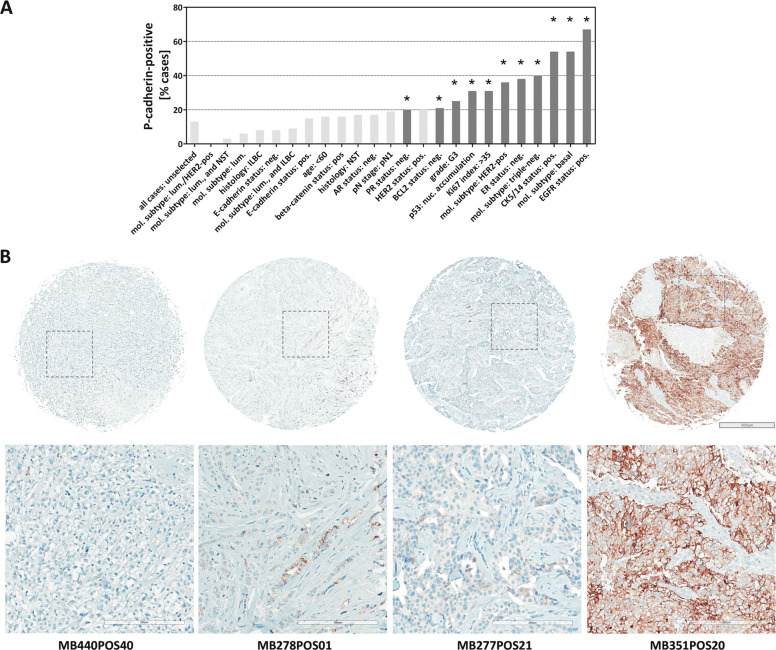
P-cadherin expression in the reference cohort of BCs compiled on TMAs. **a** Bar chart showing the frequency [%] of P-cadherin-positive (IRS ≥ 3) cases in different BC subsets. Black bars indicate phenotypic associations with *P* values < 0.050. For details see Supplementary data Table 3. **b** Shown are representative photomicrograph of P-cadherin immunohistochemical stainings. Overviews of TMA cores are shown in the upper panel. Scale bar corresponds to 500 µm. Lower panels show details at ×200 magnification. Scale bars correspond to 200 µm. From left to right, the cases correspond to an ER-positive ILBC (ID MB440POS40), an ER-positive ILBC with tubular elements (ID MB278POS01), an ER-positive BC of no special type (NST) (ID MB277POS21), and a triple-negative BC of no special type (NST) (ID MB351POS20).

Next, P-cadherin staining characteristics were compared with an independent reference cohort of invasive BCs. To this end, we utilized a series of *n* = 268 well-characterized BCs compiled on TMAs with comparatively large cores (1.5 mm diameter) considered to be representative for this purpose [22]. TMAs were subjected to P-cadherin immunohistochemistry. Using an IRS ≥ 3 as cutoff to define a positive P-cadherin status, 36/268 (13%) BCs were P-cadherin-positive (Fig. 7a). P-cadherin expression was associated with histological grade 3, TNBC, and basal markers, such as CK5/14 (all *P* < 0.001) (Supplementary data Table 3) [36]. This is consistent with previous studies [7]. TNBC was P-cadherin-positive in 21/53 (40%) cases (*P* < 0.001) (Fig. 7a). These triple-negative tumors showed strong membranous P-cadherin immunoreactivity in virtually all tumor cells corresponding to an IRS 12 (Fig. 7b, right). P-cadherin-positive TNBCs were always E-cadherin-positive, while P-cadherin negative TNBCs were partly E-cadherin negative (*P* < 0.001) (Supplementary data Table 4). Strict co-expression of P-cadherin and E-cadherin is consistent with a cell context dependent function of P-cadherin in TNBC, as reported by Ribeiro et al. [15].

ILBCs mostly lacked P-cadherin protein expression (Fig. 7b, left). P-cadherin expression was noted in only 7/84 (8%) ILBCs. In these seven cases, P-cadherin protein expression was mostly focally accentuated or showed a mosaic-like staining pattern (2× IRS 3, 2× IRS 4, 2× IRS 8, 1× IRS 12). One ILBC from the reference cohort (case MB278POS01, E-cadherin-negative, ER-positive) showed focally accentuated P-cadherin immunoreactivity associated with tubular elements (Fig. 7b, second from left). This case was originally diagnosed as mixed ductal–lobular ILBC, but was now reevaluated as another example of ILBC with tubular elements (see “Discussion” section). Only one other ILBC from the reference cohort (case MB440POS12, also E-cadherin-negative, ER-positive) showed strong P-cadherin immunoreactivity in nearly all tumor cells (Supplementary data Fig. 8, right). Compared with ILBCs from the reference cohort, P-cadherin expression was more common in the series of ILBCs with tubular elements, but was not restricted to this morphological variant (12/13 versus 7/84, *P* < 0.001) (Table 3).

**Table 3:** Comparison with ILBCs from the reference cohort.

	P-cad.		
	neg	pos	*P* value
ILBC (reference cohort)	77 (92%)	7 (8%)	
			<0.001*
ILBC with tubular elements	1 (8%)	12 (92%)	

*Pos* positive, *neg* negative.

*Fisher’s exact test.

### Reduced tumor cell proliferation in tubular elements

**Fig. 8:**
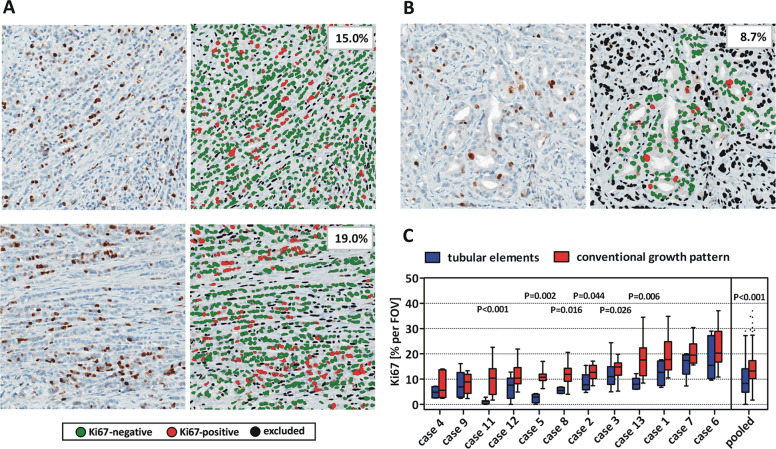
Reduced tumor cell proliferation in tubular elements. The percentage of Ki67-positive tumor cells was quantified in ILBC cells arranged in conventional growth pattern (**a**) or tubular elements (**b**). Shown are three representative fields of view (FOVs) of case 3. Ki67-stainings are shown in the left panels (×200 magnification) and Ki67 quantification by CognitionMaster software is shown in the right panels. **c** Traditional Tukey plot showing the distribution of Ki67 indices [% per FOV] in *n* = 11 ILBCs analyzed. Horizontal lines indicate the median Ki67 across multiple FOVs. Boxes indicate the interquartile ranges and whiskers indicate the 1.5-fold interquartile distance, or the minimal/maximal values, whichever is shorter. Statistical significance was determined with the Mann–Whitney test.

Eventually, we asked for the relationship between tumor growth and tubular elements. Percentages of Ki67-positive tumor cells per FOV were quantified in tubular elements and conventional growth pattern using the CognitionMaster digital pathology platform (Fig. 8a, b) [24]. ILBC cells arranged in tubular elements showed reduced proliferative activity in 6/12 (50%) cases analyzed (Fig. 8c). However, considerable intratumoral proliferative heterogeneity was common. Therefore, FOVs with either tubular elements (*n* = 85) or conventional growth pattern (*n* = 155) were pooled from all specimens. In this pooled analysis, the median Ki67 index for conventional growth pattern and tubular elements was 13.2% (interquartile range 10.2–17.3%) and 8.3% (interquartile range 4.8–14.1%), respectively (*P* < 0.001) (Fig. 8c). Accordingly, ILBC cells arranged in tubular elements display a reduced proliferative activity.

## Discussion

E-cadherin to P-cadherin switching can partially rescue AJ formation and cell–cell adhesion in the absence of E-cadherin [15, 19, 20]. E-cadherin to P-cadherin switching has been described in a variety of cancer entities, but has not been implicated in ILBC, so far [1, 7, 18]. In the present study we characterized a series of ILBCs with exceptional histomorphology, which we termed ILBCs with tubular elements. These cases were defined by noncohesive tumor cells arranged in conventional ILBC growth pattern admixed with cancerous tubular elements. All cases were ER-positive and showed complete loss of E-cadherin expression. Admixed tubular elements were E-cadherin-negative too. Most cases harbored deleterious *CDH1*/E-cadherin mutations.

Immunohistochemical analyses revealed that noncohesive tumor cells lacked beta-catenin expression. Tubular elements retained membrane-localized beta-catenin expression. This indicated a partial rescue of AJ formation. Systematic analyses of alternate type-I cadherins revealed P-cadherin expression in 12/13 (92%) cases. Strikingly, P-cadherin immunoreactivity was accentuated in tubular elements, while noncohesive tumor cells arranged in conventional ILBC growth pattern showed weaker or no immunoreactivity. This distinctive intratumoral heterogeneity or gradient argued against an interpretation of these cases as collision tumors composed of independent P-cadherin-negative and P-cadherin-positive lesions. In fact, detection of identical *CDH1*/E-cadherin mutations and matching CNAs in tumor cells microdissected from regions with pure conventional ILBC growth pattern and almost pure tubular elements proved clonal relatedness. Quantitative real-time RT-PCR showed increased *CDH3* (P-cadherin) mRNA expression in tumor cells microdissected from a region with almost pure tubular elements. However, *CDH3* mRNA was also detectable in tumor cells microdissected from a region with conventional growth pattern, suggesting a regulation of P-cadherin expression at the mRNA and protein level. In an independent cohort of invasive BCs complied on TMAs, which served as a reference, P-cadherin protein expression was associated with triple-negative nonlobular BC (TNBC). This is consistent with previous studies [7]. In ILBCs from the reference cohort, expression of P-cadherin was rare (7/84; 8%). It was more common in the series of ILBCs with tubular elements (12/13 versus 7/84, *P* < 0.001). Re-analysis of TCGA gene expression data of another independent tumor cohort also supported activation of *CDH3* (P-cadherin) mRNA expression in a subset of ILBC (4/70, 6%) [31]. Adjacent LCIS was always P-cadherin negative. Accordingly, ILBCs with tubular elements are characterized by focal E-cadherin to P-cadherin switching, which reestablishes cell adhesion and appears to facilitate focal formation of tubular elements.

BCs with mixed histology have always been of particular interest [37–42]. Mixed histology can arise through different mechanisms. Wheeler et al. and Esposito et al. have shown that E-cadherin-positive tubular BCs can focally mimic single files, a growth pattern known from classical ILBC [38, 41]. These tumors are termed tubulolobular BC. Tubulolobular BCs are E-cadherin-positive and represent a variant of tubular BC [38, 41, 43]. Qureshi et al. have described BCs with E-cadherin-positive tubules and E-cadherin-negative single files [39]. Most likely, these tumors were examples of mixed ductal–lobular BCs sensu stricto (collision tumors). McCart-Reed et al. have exemplified that secondary *CDH1*/E-cadherin mutations can induce E-cadherin negative subclones with ILBC-like morphology within BCs of NST [42]. The present study adds another explanation for mixed-appearing histology. E-cadherin-negative tubules can arise in *CDH1*-defective ILBCs as a consequence of a focal E-cadherin to P-cadherin switching (or conversion from E-cadherin to P-cadherin). These cases should probably be classified as a morphological variant of ILBC. In fact, focal E-cadherin-negative tubules are well-known in ILBC and they have never precluded the diagnosis of ILBC [44]. However, their immunophenotypic properties and relatedness to P-cadherin expression has remained unknown, so far. Accordingly, we term these cases ILBC with tubular elements. We avoid the terminus tubulolobular BC, which is already reserved for tubulolobular BCs with E-cadherin-positive single files, such as described by Wheeler et al. and by Esposito et al. [38, 41]. In routine diagnostics, optional ancillary immunohistochemical studies can support the differential diagnosis between tubulolobular BC, mixed ductal–lobular BC and ILBC with tubular elements (Table 4).

**Table 4:** BCs with mixed-appearing histology.

	Noncohesive tumor cells	Tubules	References
Tubulolobular BC	E-cad. pos	E-cad. pos	Wheeler et al. [38], Esposito et al. [41], Kuroda et al. [40]
Mixed ductal–lobular BC (sensu stricto)	E-cad. neg	E-cad. pos	Qureshi et al. [39], McCart-Reed et al. [42]
ILBC with tubular elements	E-cad. neg	E-cad. neg and P-cad. pos	Present study

The present study may also contribute to a better understanding of the tumorbiology of ILBC. Inactivation of E-cadherin is the key tumorigenic driver of ILBC [8, 9, 12]. E-cadherin to P-cadherin switching may be dynamically regulated and could be reversible. If so, the true frequency of E-cadherin to P-cadherin switching in patients over time would be difficult or impossible to determine. However, P-cadherin may transiently substitute E-cadherin and may inhibit tumor growth in this particular cellular context [15]. Strikingly, significantly reduced tumor cell proliferation was noted in tubular elements in some, but not all ILBC specimens. Accordingly, E-cadherin to P-cadherin switching may be involved in tumor dormancy in ILBC, but this is beyond the scope of the present work.

In summary, E-cadherin to P-cadherin switching occurs in a subset of ILBCs and correlates with mixed-appearing growth pattern characterized by E-cadherin-negative tubular elements. This has implications for histological BC diagnostics and contributes to the tumorbiological understanding of ILBC.

## Supplementary information

Supplementary Material

OA Payment Form
