# Supplementary material for: E-cadherin to P-cadherin switching in lobular breast cancer with tubular elements
Source: Mod Pathol. 2020 Jun 22;33(12):2483–98. doi: 10.1038/s41379-020-0591-3 (PMC7685979; doi:10.1038/s41379-020-0591-3)

Christgen *et al.* 2020

E-cadherin to P-cadherin switching in lobular breast cancer with tubular elements

Supplementary Material

1. List of Supplemental data

2. Supplemental Data Table 1. Antibodies used for immunohistochemistry.

3. Supplemental Data Table 2. Mutational analysis, NGS details.

4. Supplemental Data Table 3. BC reference cohort, TMA details.

5. Supplemental Data Table 4. Co-expression of P-cadherin and E-cadherin in TNBC.

6. Supplemental Data Figure 1. Histomorpholoy of tubular elements (overview).

7. Supplemental Data Figure 2. cnLOH or LOH of chromosome 16q22.1 in cases 3, 5, 6 and 13.

8. Supplemental Data Figure 3. CNAs in ILBC with tubular elements and adjacent conventional ILBC

9. Supplemental Data Figure 4. Positive controls for P-, N- and R-cadherin immunohistochemistry.

10. Supplemental Data Figure 5. Budding of P-cadherin-negative ILBC cells (case 10).

11. Supplemental Data Figure 6. Additional representative immunohistochemical stainings (case 13).

12. Supplemental Data Figure 7. Additional representative immunohistochemical stainings (case 11).

13. Supplemental Data Figure 8. P-cadherin expression in ILBCs from the TMA reference cohort.

2. Supplemental Data Table 1

Supplemental Data Table 1

Antibodies used for immunohistochemical analyses

A) ILBC with tubular elements

antigen	study	antibody	species	source	dilution	antigenic retrieval	detection system	scoring	cutoff
ER	ILBC-TE	clone SP1	rabbit	Ventana	undiluted, ready-to use solution	CC1 mild protocol (Ventana)	ultraView DAB Kit (Ventana)	semiquantitative, 0-100	10
PR	ILBC-TE	clone 1E2	rabbit	Ventana	undiluted, ready-to use solution	CC1 mild protocol (Ventana)	ultraView DAB Kit (Ventana)	semiquantitative, 0-100	10
HER2	ILBC-TE	clone 4B5	mouse	Ventana	undiluted, ready-to use solution	CC1 mild protocol (Ventana)	ultraView DAB Kit (Ventana)	0, 1+, 2+, 3+	2+, 3+
Ki67	ILBC-TE	clone 30-9	rabbit	Ventana	undiluted, ready-to use solution	CC1 mild protocol (Ventana)	ultraView DAB Kit (Ventana)	semiquantitative, 0-100	n.a.
E-cadherin	ILBC-TE	clone ECH-6	mouse	Zytomed	1:100	CC1 mild protocol (Ventana)	ultraView DAB Kit (Ventana)	Remmele IRS	0
beta-catenin	ILBC-TE	clone 14	mouse	BD Transduction Laboratories	1:75	CC1 mild protocol (Ventana)	ultraView DAB Kit (Ventana)	Remmele IRS	0
p120-catenin	ILBC-TE	clone 98	mouse	BD Transduction Laboratories	1:250	CC1 mild protocol (Ventana)	ultraView DAB Kit (Ventana)	Remmele IRS	>/=3, localization
P-cadherin	ILBC-TE	clone 56	mouse	BD Transduction Laboratories	1:100	CC1 mild protocol (Ventana)	CC1 mild protocol (Ventana)	Remmele IRS	>/=3
R-cadherin	ILBC-TE	clone D9	mouse	Santa Cruz	1:50	CC1 mild protocol (Ventana)	CC1 mild protocol (Ventana)	Remmele IRS	>/=3
N-cadherin	ILBC-TE	clone 3B9	mouse	Thermo Fisher Scientific	1:100	CC1 mild protocol (Ventana)	CC1 mild protocol (Ventana)	Remmele IRS	>/=3

B) TMA reference cohort

antigen	study	antibody	species	source	dilution	antigenic retrieval	detection system	scoring	cutoff
ER	TMA	clone SP1	rabbit	Neomarkers, MEDAC	1:100	citric acid, pH 6.0, pressure cooker, 125 °C	ZytoChem-Plus HRP Kit (Zytomed)	Remmele IRS	3
PR	TMA	clone PgR636	mouse	Dako	1:100	citric acid, pH 6.0, pressure cooker, 125 °C	ZytoChem-Plus HRP Kit (Zytomed)	Remmele IRS	3
AR	TMA	clone AR441	mouse	Dako	1:40	CC1 mild protocol (Ventana)	ultraView DAB Kit (Ventana)	Remmele IRS	3
BCL2	TMA	clone 124	mouse	Dako	1:100	CC1 mild protocol (Ventana)	ultraView DAB Kit (Ventana)	Remmele IRS	3
HER2	TMA	clone 4B5	mouse	Ventana	undiluted, ready-to use solution	CC1 mild protocol (Ventana)	ultraView DAB Kit (Ventana)	0, 1+, 2+, 3+	2+, 3+
CK5/14	TMA	clones XM26 and LL002	mouse	Zytomed	1:200	citric acid, pH 6.0, pressure cooker, 125 °C	ZytoChem-Plus HRP Kit (Zytomed)	Remmele IRS	3
EGFR	TMA	clone 2.1E1	mouse	Zytomed	1:600	Fast Enzyme Solution (Zytomed)	ZytoChem-Plus HRP Kit (Zytomed)	Remmele IRS	3
p53	TMA	clone DO-7	mouse	Novocastra	1:100	CC1 mild protocol (Ventana)	ultraView DAB Kit (Ventana)	semiquantitative, 0-100	>95
E-cadherin	TMA	clone 4A2C7	mouse	Invitrogen	1:50	citric acid, pH 6.0, pressure cooker, 125 °C	ZytoChem-Plus HRP Kit (Zytomed)	semiquantitative 0-100	0
beta-catenin	TMA	clone #14	mouse	BD Transduction Laboratories	1:100	citric acid, pH 6.0, pressure cooker, 125 °C	ZytoChem-Plus HRP Kit (Zytomed)	Remmele IRS	3

Ki67	TMA	clone 30-9	rabbit	Ventana	undiluted, ready-to use solution	CC1 mild protocol (Ventana)	ultraView DAB Kit (Ventana)	semiquantitative, 0-100	10, 25, 35
P-cadherin	TMA	clone 56	mouse	BD Transduction Laboratories	1:100	CC1 mild protocol (Ventana)	CC1 mild protocol (Ventana)	Remmele IRS	>/=3

3. Supplemental Data Table 2

Supplemental Data Table 2

NGS details

case	format	histology	pre-preparation	reads	coverage	gene	mut. status	freq.
A) primary tumors: customized CDH1 panel								
1	RS	ILBC with tubular elements	microdissected	222775	4790	*CDH1*	p.H97I_fs*20	0,61
2	RS	ILBC with tubular elements	bulk	1891618	57331	*CDH1*	p.F86S_fs12	0,42
3	RS	ILBC with tubular elements, ovarian met.	microdissected region with only tubular elements	804829	25954	*CDH1*	p.S9*	0,75
4	NB	ILBC with tubular elements	bulk	692796	22824	*CDH1*	p.E841*	0,37
5	RS	ILBC with tubular elements	microdissected	707935	22699	*CDH1*	p.Q23*	0,38
6	NB	ILBC with tubular elements	bulk	936629	23332	*CDH1*	wild-type	ina
7	NB	ILBC with tubular elements	bulk	725175	23535	*CDH1*	p.Q177*	0,47
8	NB	ILBC with tubular elements	bulk	35952	857	*CDH1*	p.E445*	0,61
9	RS	ILBC with tubular elements	microdissected	251137	8181	*CDH1*	splicing	0,28
10	RS	ILBC with tubular elements	bulk	47660	1155	*CDH1*	p.T295I	0,04
11	RS	ILBC with tubular elements	bulk	38333	1060	*CDH1*	p.P537R_fs*20	0,31
12	NB	ILBC with tubular elements	bulk	101856	3141	*CDH1*	p.Q23*	0,43
13	RS	ILBC with tubular elements	bulk	524266	18456	*CDH1*	wild-type	ina
B) additional tumors/lesions: customized CDH1 panel								
1	RS	2nd tumor, ipsilateral, ILBC with classic growth pattern	microdissected	122103	3287	*CDH1*	p.H97I_fs*20	0,27
1	RS	3nd tumor, ipsilateral, ILBC with solid growth pattern	microdissected	198147	3462	*CDH1*	p.H97I_fs*20	0,58
1	RS	adjacent LCIS	microdissected	273633	6170	*CDH1*	p.H97I_fs*20	0,22
3	RS	ILBC with conventional growth pattern, ovarian met.	microdissected region with only conventional growth pattern	796760	25914	*CDH1*	p.S9*	0,70
4	NB	2nd tumor, ipsilateral, ILBC with classic growth pattern	bulk	821730	27473	*CDH1*	p.E841*	0,10
4	NB	3rd tumor, contralateral, ILBC with classic growth pattern	bulk	798321	26415	*CDH1*	p.Y523*	0,36
5	RS	2nd tumor, ipsilateral, ILBC with trabecular growth pattern	microdissected	635777	20215	*CDH1*	p.Q23*	0,12
9	RS	2nd tumor, ipsilateral, ILBC with classic growth pattern	bulk	243778	7545	*CDH1*	splicing	0,28
C) case 3: additional Oncomine comprehensive assay								
3	RS	ILBC with tubular elements, ovarian met.	microdissected region with only tubular elements	5562786	1587	*TSC1*<br>*RNF43*	p.G568*<br>p.A193Pfs*10	0,80<br>0,33
3	RS	ILBC with conventional growth pattern, ovarian met.	microdissected region with only conventional growth pattern	9907247	2856	*TSC1*<br>*RNF4*3<br>*PTEN*	p.G568*<br>p.A193Pfs*10<br>p.V271L	0,60<br>0,39<br>0,05

4. Supplemental Data Table 3

Supplemental Data Table 3

Association of P-Cadherin with clinicopathological characteristics, TMA cohort

		all cases		P-cadherin-pos.		P-cadherin-neg.					
		number	percent	number	percent	number	percent	P value		test	note
all cases		268	100	36	13	232	87				
age								0.368		FET	
	<60	120	45	19	16	101	84				
	>/=60	148	55	17	11	131	88				
lesion type								0.051		FET	PT vs DOM
	PT	182	68	26	14	156	86				
	LRT	36	13	8	22	28	78				
	DOM	50	19	2	4	48	96				
pT stage								0.805		FET	pT1/2 vs pT3/4
	pT1/2	157	59	23	15	134	85				
	pT3/4	41	15	5	12	36	88				
	n.a.	20	7	3	15	17	85				
	ina.	50	19	5	10	45	90				
pN stage								0.278		FET	pN0 vs pN1+
	pN0	98	37	12	12	86	88				
	pN1+	70	26	13	19	57	81				
	n.a.	50	19	9	18	41	82				
	ina.	50	19	2	4	48	96				
histology								0.082		FET	NST vs lobular
	NST	175	65	29	17	146	83				
	lobular	84	31	7	8	77	92				
	mucinous	8	3	0	0	8	100				
	tubular	1	1	0	0	1	100				
grade								<0.001	*	CSTT	
	G1	9	3	0	0	9	100				
	G2	148	55	10	7	138	93				
	G3	98	37	25	25	73	75				
	n.a.	13	5	1	8	12	92				
ER status								<0.001	*	FET	
	pos.	203	76	11	5	192	95				
	neg.	65	24	25	38	40	62				
PR status								<0.001	*	FET	
	pos.	126	47	7	6	119	94				
	neg.	142	53	29	20	113	80				

AR status									
	pos.	62	23	6	10	56	90	0.211		FET
	neg.	160	60	27	17	133	83			
	n.a.	46	17	3	6	43	94			
BCL2 status								<0.001	*	FET
	pos.	118	44	4	3	114	97			
	neg.	142	53	30	21	112	79			
	n.a.	8	3	2	25	6	75			
HER2 status								0.323		FET
	0/1+	241	90	32	13	209	87			
	2+/F-	7	3	0	0	7	100			
	2+/F+	0	0	0	0	0	0			
	3+	20	7	4	20	16	80			
CK5/14 status								<0.001	*	FET
	pos.	22	8	12	54	10	46			
	neg.	243	91	23	9	220	91			
	n.a.	3	1	1	33	2	67			
EGFR status								0.046	*	FET
	pos.	3	1	2	67	1	33			
	neg.	263	98	33	12	230	88			
	n.a.	2	1	1	50	1	50			
P53 nuc. accumulation								0.002	*	FET
	pos.	35	13	11	31	24	69			
	neg.	227	85	23	10	204	90			
	n.a.	6	2	2	33	4	67			
E-cadherin status								0.083		FET
	pos.	170	63	26	15	144	85			
	neg.	91	34	7	8	84	92			
	n.a.	7	3	3	43	4	57			
Beta-catenin status								0.051		FET
	pos.	180	67	29	16	151	84			
	neg.	85	32	6	7	79	93			
	n.a.	3	1	1	33	2	67			
Ki67 index								<0.001	*	CSTT
	0-10	84	31	6	7	78	93			
	11-25	103	38	10	10	93	90			
	26-35	26	10	3	11	23	89			
	36-100	55	21	17	31	38	69			
molecular subtype								<0.001	*	CSTI
	luminal A/B	195	73	11	6	184	94			

luminal / HER2-pos.	9	3	0	0	9	100			
HER2-pos.	11	4	4	36	7	64			
basal	22	8	12	54	10	46			
undefined	31	12	9	29	22	71			
							<0.001	*	CSTI	
triple-neg.	53	20	21	40	32	60	0.127		FET	luminal NST vs ILBC
luminal, and NST	113	42	4	3	109	97				
luminal, and ILBC	78	29	7	9	71	91				

5. Supplemental Data Table 4

Supplemental Data Table 4

P-cadherin and E-cadherin expression in TNBC

		P-cad.				
		pos		neg		P value
E-cad.	pos	18	(34%)	22	(41%)	
	neg	0	(0%)	10	(19%)	0.009*
	na	3	(6%)	0	(0%)	

* Fisher's exact test

6. Supplemental Data Figure 1

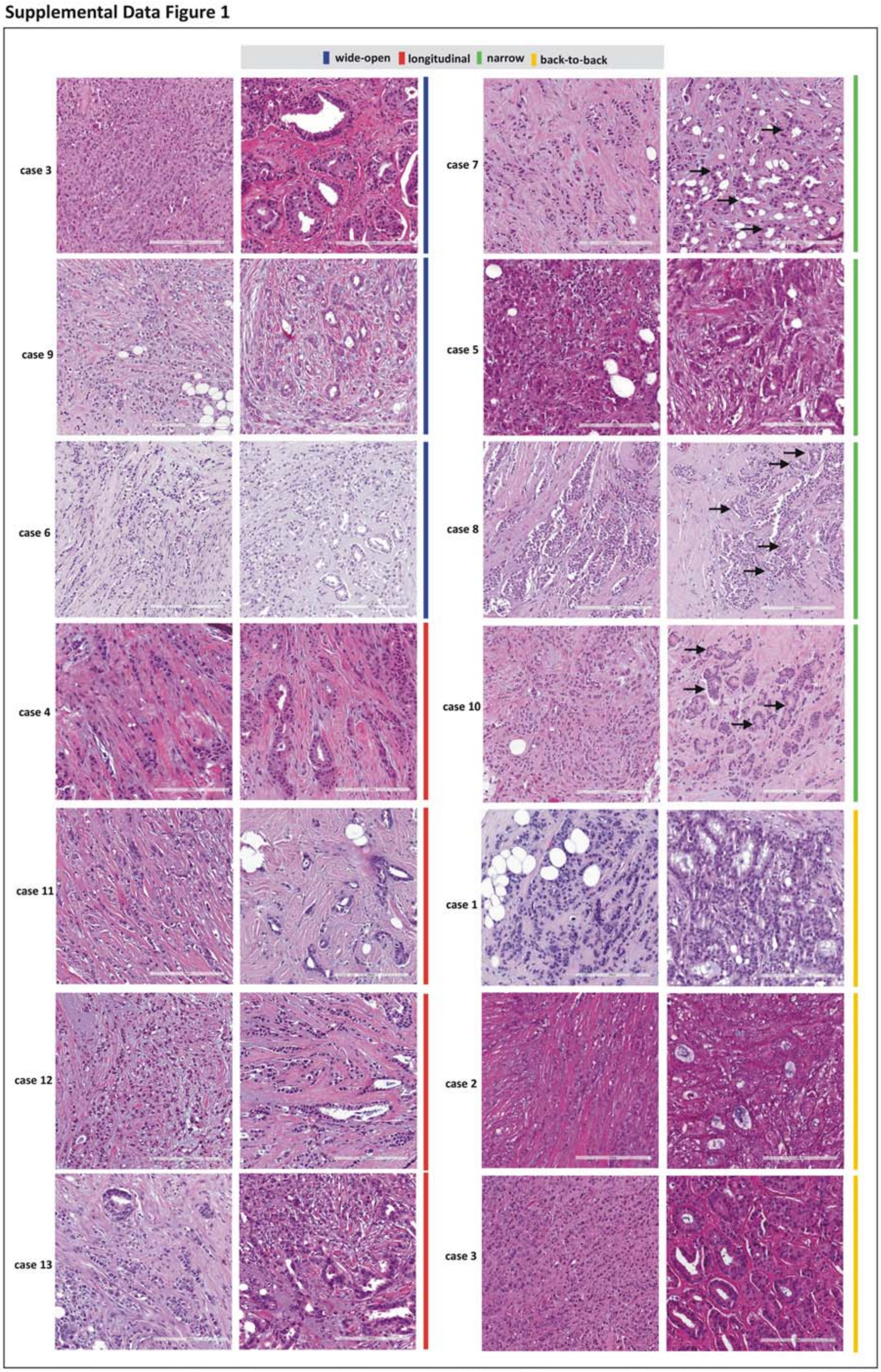

7. Supplemental Data Figure 2

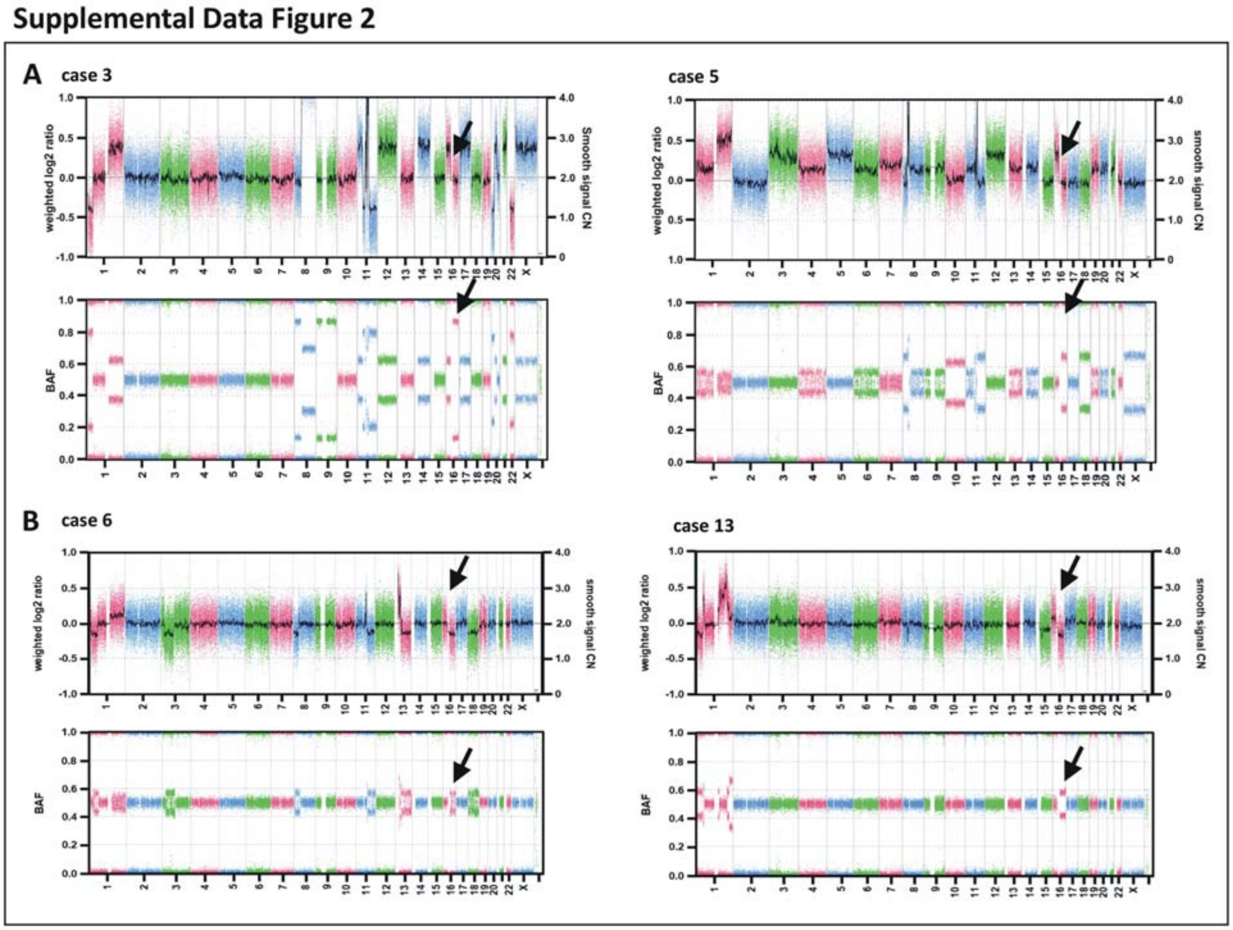

8. Supplemental Data Figure 3

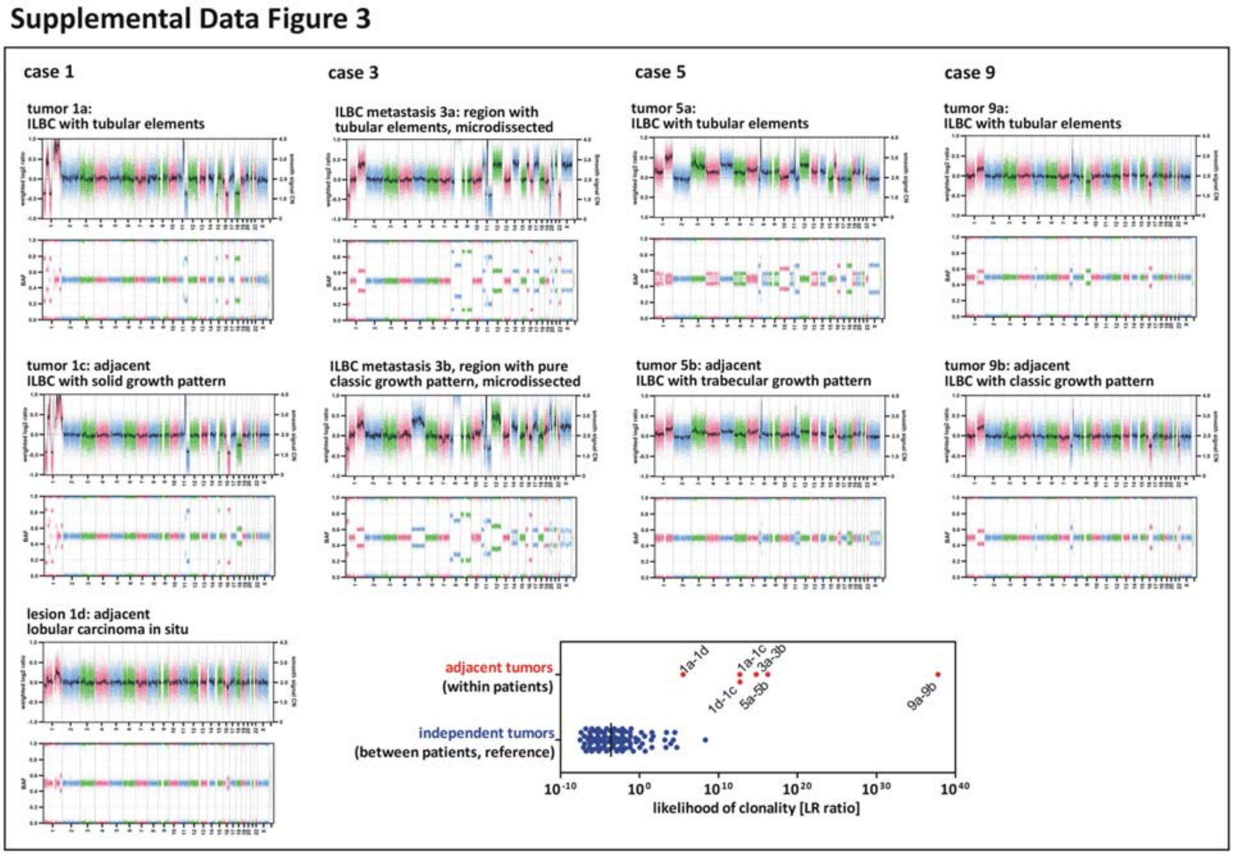

9. Supplemental Data Figure 4

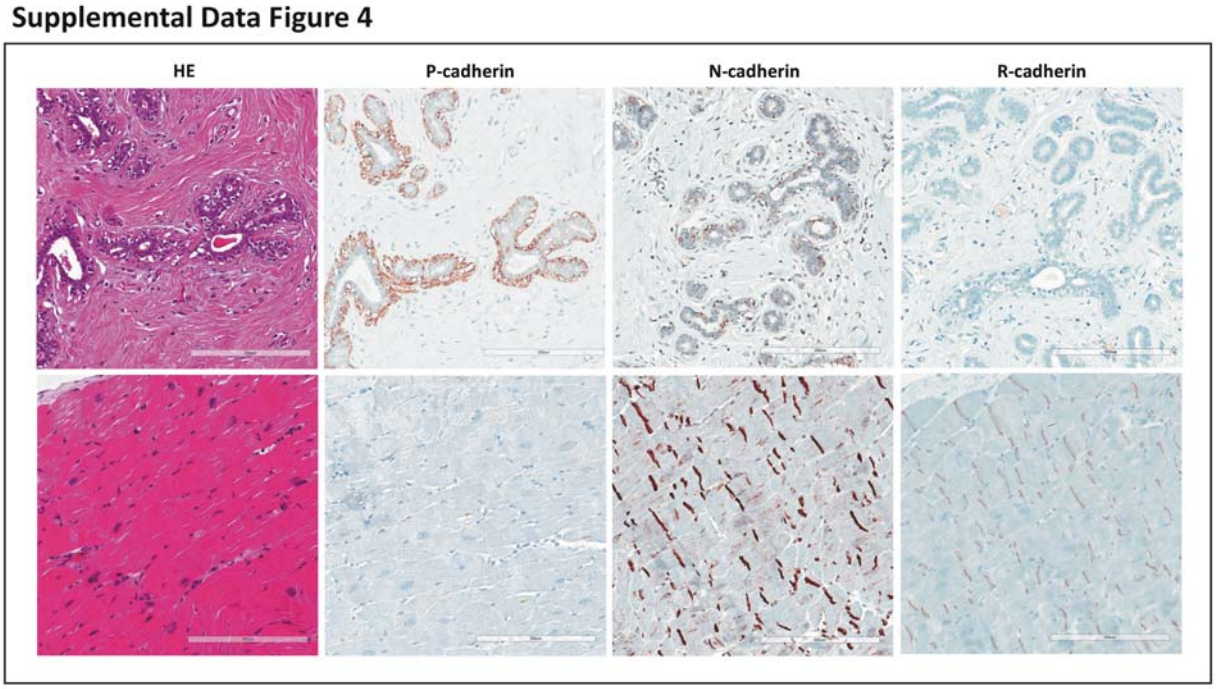

10. Supplemental Data Figure 5

Supplemental Data Figure 5

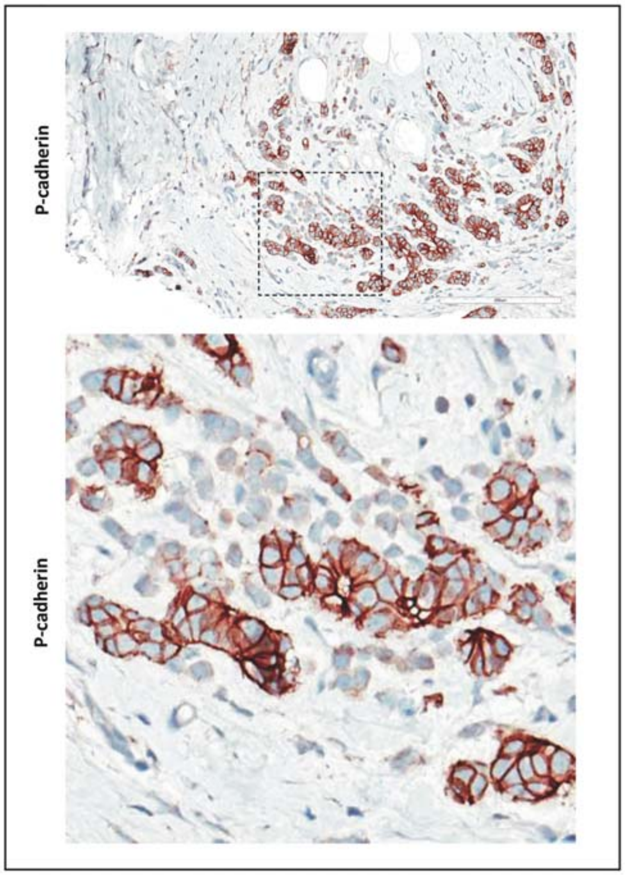

11. Supplemental Data Figure 6

Supplemental Data Figure 6

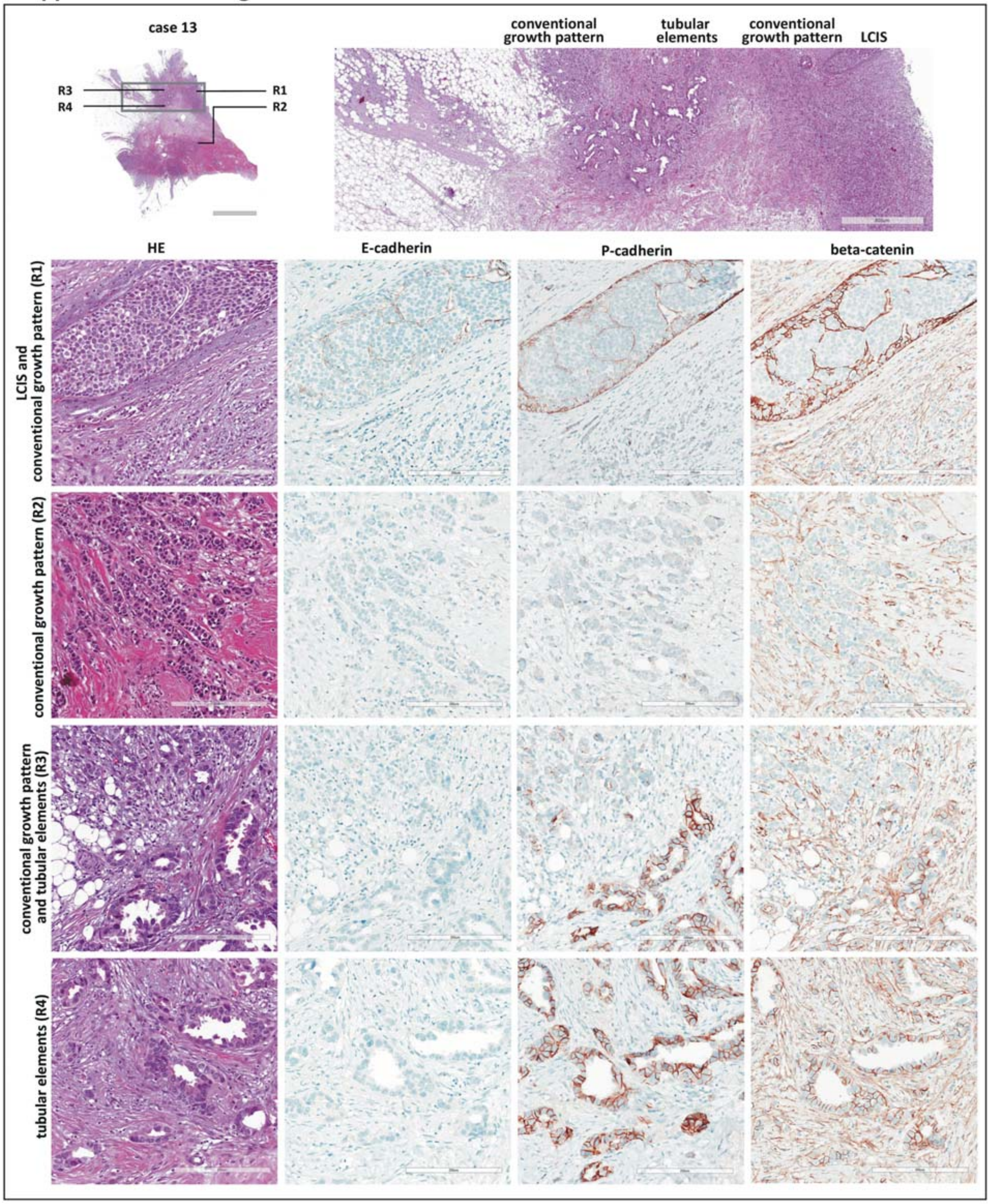

12. Supplemental Data Figure 7

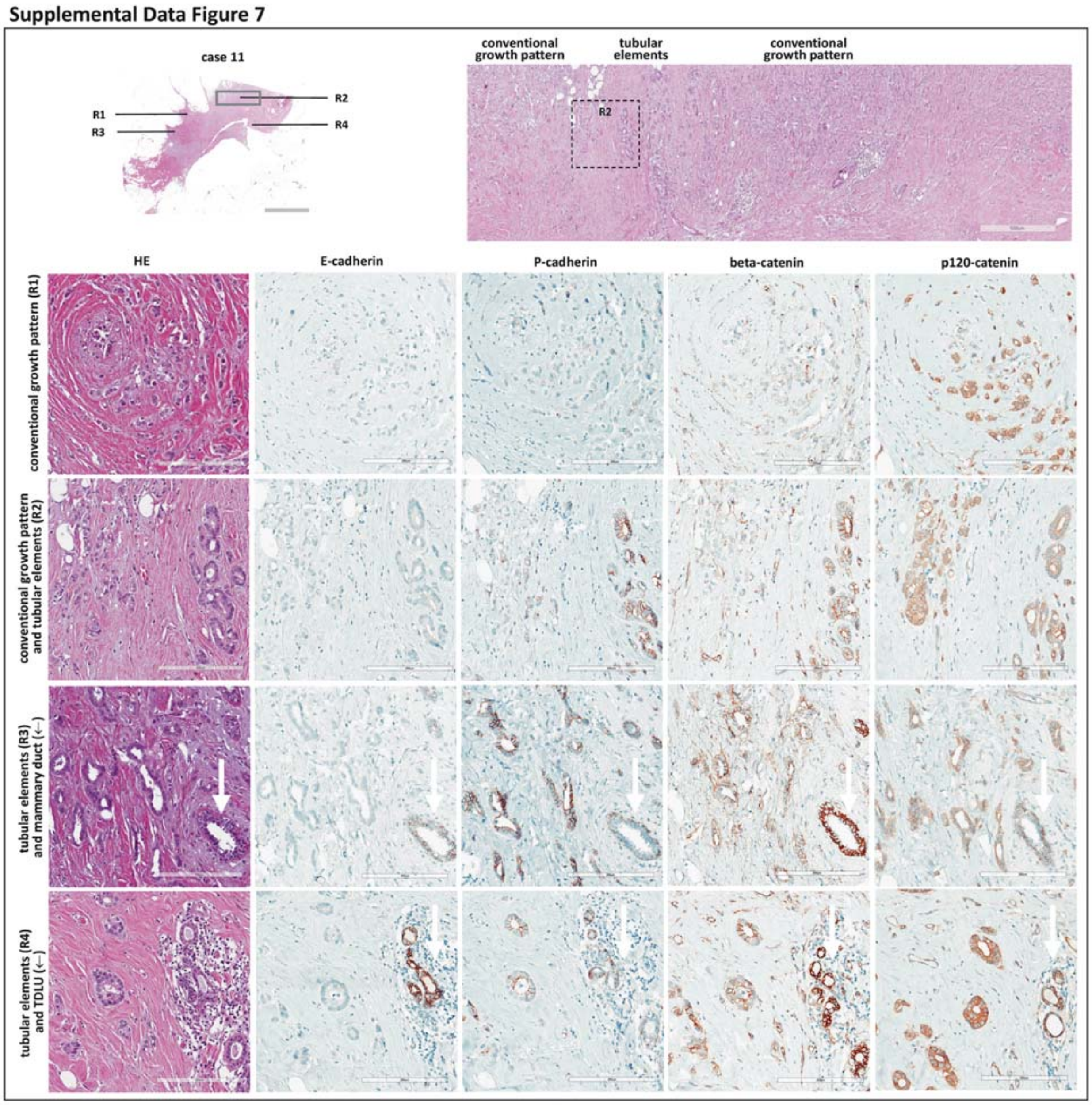

13. Supplemental Data Figure 8

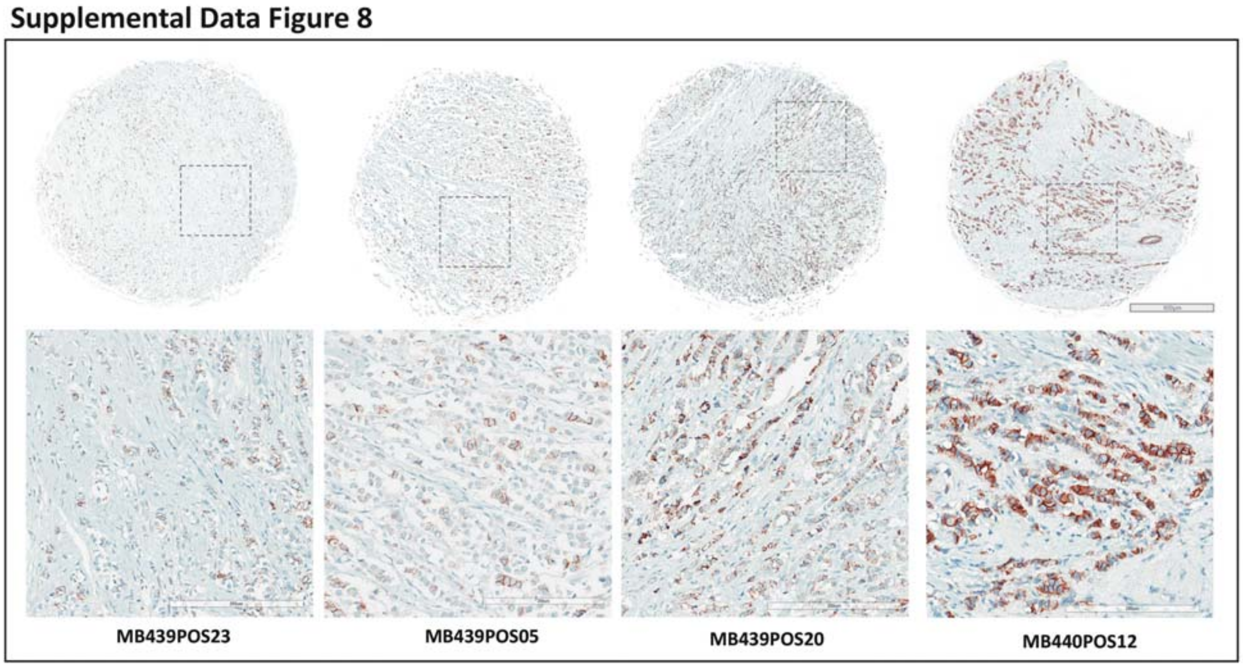

Supplement: Supplementary file 1 — Supplementary Material [file 41379_2020_591_MOESM1_ESM.pdf]
